# Precision in Pulmonary Embolism Diagnosis: Leveraging D-dimer Levels With Computed Tomography Pulmonary Angiography (CTPA) Insights

**DOI:** 10.7759/cureus.67765

**Published:** 2024-08-25

**Authors:** Arularasu P, Gokulakrishnan Sekar, Nalini Jayanthi Nagesh

**Affiliations:** 1 Respiratory Medicine, SRM Medical College Hospital and Research Centre, Chennai, IND

**Keywords:** lobar arteries, central embolism, computed tomography pulmonary angiography, d-dimer levels, pulmonary embolism

## Abstract

Introduction

Pulmonary embolism (PE) remains a critical condition requiring timely diagnosis and treatment. The use of D-dimer, a fibrin degradation product, as a biomarker, combined with computed tomography pulmonary angiography (CTPA), is a common practice in diagnosing PE.

Aim

This study aims to increase diagnostic accuracy for PE by relating the D-dimer levels to the findings on CTPA. Specifically, it aims to calculate the sensitivity and specificity of D-dimer levels against CTPA results and also establish the association of D-dimer levels with the location of the PE.

Methods

This retrospective analysis was conducted at a tertiary care hospital, including patients who underwent CTPA and had D-dimer levels recorded over a one-year period. The total sample size was 124. D-dimer levels were categorized into four groups based on CTPA findings: Category 0 (no PE), Category I (peripheral PE), Category II (PE in lobar arteries), and Category III (central embolisms in the pulmonary trunk or arteries). Statistical analyses were performed to evaluate the correlation between D-dimer levels and CTPA findings, including sensitivity, specificity, positive predictive value (PPV), and negative predictive value (NPV).

Results

The study found that Category 0 (no PE) had an average D-dimer of 3.6 mg/L, Category I (peripheral PE) had 4.3 mg/L, Category II (PE in lobar arteries) had 3.6 mg/L, and Category III (central embolisms) had 7.1 mg/L. The sensitivity of D-dimer in predicting PE was 1.0, and the specificity was 0.2. The PPV was 0.3208, and the NPV was 1.0. These findings indicate a significant correlation between elevated D-dimer levels and the presence of PE.

Conclusion

Integrating D-dimer levels with CTPA findings can improve diagnostic accuracy and efficiency for PE. Establishing reliable D-dimer cutoff values may help clinicians better stratify patient risk and make informed decisions about the need for imaging, thereby optimizing resource utilization and minimizing unnecessary CTPA scans. This study highlights the potential benefits of combining biomarker analysis with imaging results in the clinical management of PE.

## Introduction

Pulmonary embolism (PE) is a critical and potentially life-threatening condition that necessitates prompt and accurate diagnosis. PE occurs when a blood clot obstructs the pulmonary arteries, impairing blood flow and potentially damaging lung tissue. The clinical presentation of PE can vary widely, from asymptomatic cases to severe, life-threatening scenarios. Early diagnosis and treatment are crucial to improving patient outcomes and reducing mortality rates [1].

D-dimer is a fibrin degradation product present in the blood after a blood clot is degraded by fibrinolysis. It is typically elevated in thromboembolic events, including PE. The utility of D-dimer as a diagnostic tool lies in its high sensitivity, making it a useful initial test to rule out PE in patients with low to moderate clinical probability [2]. Elevated D-dimer levels can indicate the presence of an abnormal blood clot but are not specific to PE, as they can be elevated in other conditions such as inflammation, trauma, or recent surgery. This limitation often necessitates further confirmatory testing, such as imaging studies, to accurately diagnose PE [3].

Computed tomography pulmonary angiography (CTPA) is considered the gold standard imaging technique for diagnosing PE. CTPA provides detailed images of the pulmonary arteries and can directly visualize blood clots obstructing these vessels. The high specificity of CTPA makes it a valuable tool for confirming PE diagnosis in patients with elevated D-dimer levels or other clinical signs of PE [4]. Despite its diagnostic accuracy, the accessibility, cost, and exposure to radiation associated with CTPA necessitate its judicious use, particularly in emergency settings [5].

Understanding the relationship between D-dimer levels and CTPA findings is crucial for optimizing the diagnostic process for PE. By identifying reliable D-dimer cutoff values that correlate with the presence and severity of PE, clinicians can make more informed decisions about when to utilize CTPA, potentially reducing unnecessary imaging and associated healthcare costs. This approach aims to enhance diagnostic accuracy while minimizing patient exposure to invasive procedures and radiation [6].

This study aims to investigate the correlation between D-dimer levels and CTPA findings in patients suspected of having PE. Specifically, it seeks to establish D-dimer cutoff values <0.5 mg/L that accurately predict PE presence and assess the sensitivity and specificity of D-dimer levels in conjunction with CTPA results. The findings are expected to provide insights into the potential for integrating D-dimer testing with CTPA to improve the efficiency and effectiveness of PE diagnosis [7].

In conclusion, while D-dimer is a highly sensitive marker for thromboembolic events, its lack of specificity necessitates further diagnostic confirmation through imaging studies such as CTPA. By exploring the correlation between D-dimer levels and CTPA findings, this study aims to refine diagnostic protocols and improve patient outcomes in the management of PE.

## Materials and methods

Study design and setting

This retrospective case study was conducted at a tertiary care hospital and focused on patients who underwent CTPA with recorded D-dimer levels from May 2023 to May 2024. The study protocol received approval from the institutional ethics committee, which determined that informed consent was not required due to the retrospective nature of the study. The primary goal was to explore the relationship between D-dimer levels and CTPA results to enhance the diagnostic accuracy for PE. The study population included all patients who had CTPA within the designated year, totaling 124 individuals. These patients were divided into four categories based on their CTPA findings: Category 0 for no PE, Category I for peripheral PE, Category II for PE in lobar arteries, and Category III for central embolisms in the pulmonary trunk or arteries. The inclusion criteria comprised patients aged 18 and above, those who underwent CTPA during the specified period, those with complete medical records, including CTPA reports and D-dimer test results, and patients with a radiologically confirmed diagnosis of PE based on CTPA findings. The exclusion criteria were set to exclude patients with incomplete CTPA reports or missing D-dimer test results.

Data collection and statistical analysis

Data collection involved gathering D-dimer levels and CTPA results from hospital records. D-dimer levels were measured using standardized assays, and CTPA reports were evaluated based on established radiological criteria to ascertain the presence or absence of PE. Statistical analysis was conducted using Microsoft Excel (Microsoft Corporation, Redmond, Washington) and IBM SPSS Statistics for Windows, Version 20 (Released 2011; IBM Corp., Armonk, New York). Descriptive statistics, such as means and standard deviations, were computed to provide a summary of the data.

## Results

The study categorized 124 patients who underwent CTPA and D-dimer testing. The mean age of the 124 patients is approximately 52 ± 17.2 SD years. Approximately 57 of the patients were female, while 67 were male. The correlation between D-dimer levels and the presence of PE was assessed using sensitivity, specificity, positive predictive value (PPV), and negative predictive value (NPV). The results indicated a significant correlation between elevated D-dimer levels and the presence of PE. The sensitivity of the D-dimer test is 1.0, and its specificity is 0.2, with a PPV of 0.32 and an NPV of 1.0. These metrics indicate that although D-dimer is highly sensitive for detecting PE, its low specificity implies that it is less adept at differentiating PE from other conditions that also raise D-dimer levels. The PPV indicates that 32% of patients with elevated D-dimer levels had PE, whereas the NPV shows that patients with low D-dimer levels were correctly identified as not having PE.

**Figure 1:**
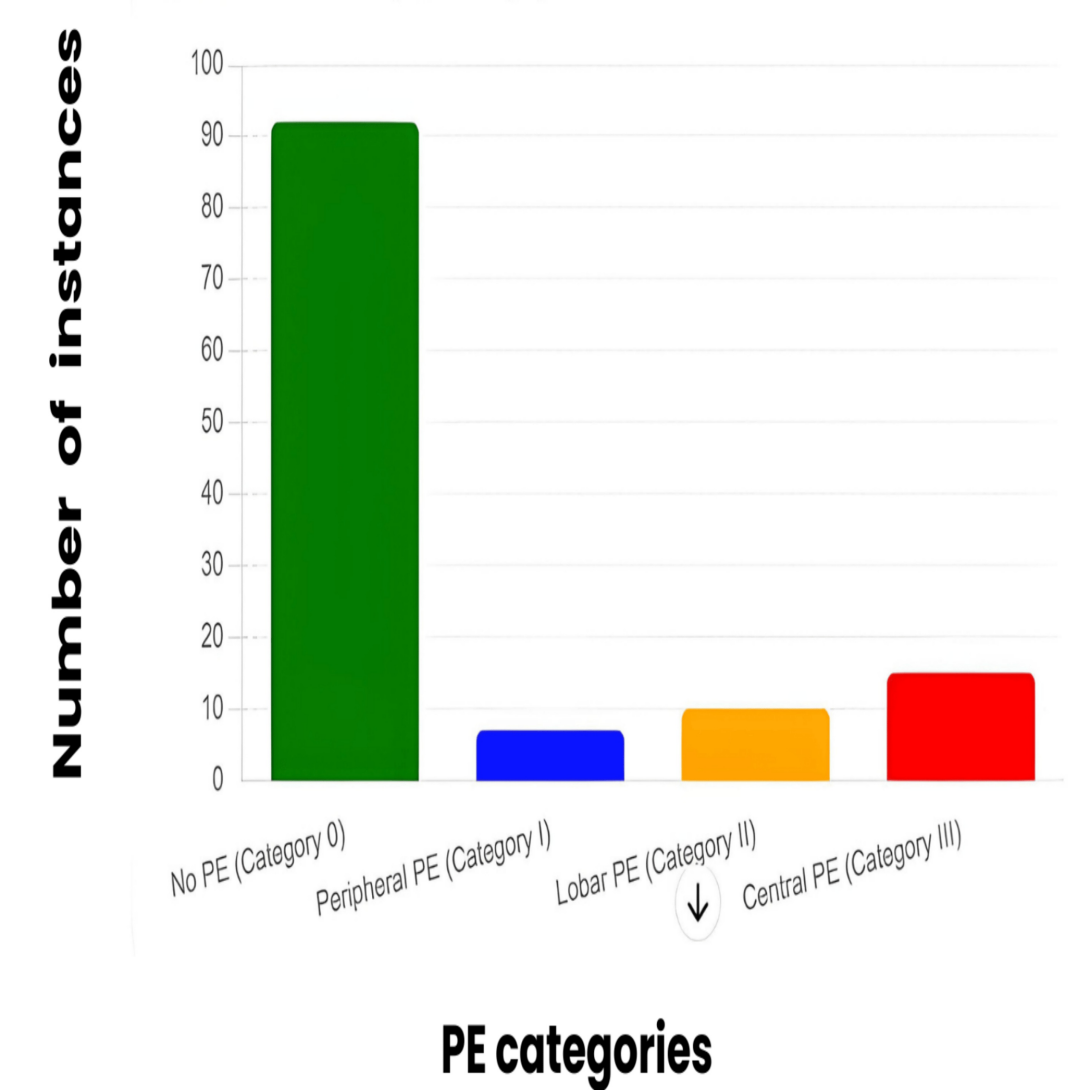
Distribution of pulmonary embolism instances across different categories. Based on the provided details regarding the CTPA results and their classification into four categories of pulmonary embolism (PE), here is a revised legend for the image: (1) no pulmonary embolism (Category 0) represents instances where no pulmonary embolism is detected, totaling 92 cases; (2) peripheral pulmonary embolism (Category I) indicates seven instances where pulmonary embolism is found in the peripheral regions of the lungs; (3) lobar pulmonary embolism (Category II) includes 10 cases where the embolism is located in the lobar branches of the pulmonary arteries; (4) central pulmonary embolism (Category III) reports 15 instances where the pulmonary embolism is located in the central pulmonary trunk or arteries.

The CTPA results of the patients were categorized into four groups based on their PE status: Category 0 (no PE), Category I (peripheral PE), Category II (PE in lobar arteries), and Category III (central embolisms in the pulmonary trunk or arteries). The distribution of D-dimer levels and the number of instances in each category were analyzed. Figure 1 categorizes the instances of PE into four groups based on their location within the lungs: Category 0 indicates 92 cases with no PE, Category I includes seven instances of peripheral PE, Category II has 10 cases of lobar PE, and Category III reports 15 instances of central PE.

Based on 124 instances, the following is the distribution of D-dimer levels across different PE categories. No PE (Category 0): Of 124 instances, 92 were classified as having no PE, with an average D-dimer level of 3.6 mg/L. Peripheral PE (Category I): This category includes seven instances, with an average D-dimer level of 4.3 mg/L. Lobar PE (Category II): There were 10 instances in this category, with an average D-dimer level of 3.6 mg/L, identical to the levels observed in Category 0. Central PE (Category III): This category had 15 instances, with a significantly higher average D-dimer level of 7.1 mg/L (Table 1 ).

**Table 1:** D-dimer levels by pulmonary embolism categories. Category 0: no pulmonary embolism; Category I: peripheral pulmonary embolism; Category II: lobar pulmonary embolism; Category III: central pulmonary embolism.

Category	Instances	D-dimer
Category 0: No PE	92	3.6
Category I: Peripheral PE	7	4.3
Category II: Lobar PE	10	3.6
Category III: Central PE	15	7.1

## Discussion

This retrospective study investigated the correlation between D-dimer levels and the presence of PE, as determined by CTPA. The results demonstrated a significant correlation between elevated D-dimer levels and the severity of PE. Higher D-dimer levels were notably associated with more central and severe embolisms (Category III), while lower levels were observed in patients with no PE (Category 0). These findings align with previous research indicating that elevated D-dimer levels can serve as a valuable indicator of the presence and severity of PE [1,2].

The sensitivity of D-dimer in predicting PE was found to be 1.0, indicating that D-dimer is highly effective in identifying patients with PE. However, the specificity was 0.2, which is relatively low, reflecting the D-dimer's inability to distinguish between PE and other conditions that elevate D-dimer levels [3,4]. The high sensitivity suggests that D-dimer is a reliable screening tool for ruling out PE, especially in patients with low clinical probability. However, due to its low specificity, it should not be used as a standalone diagnostic tool without confirmatory imaging such as CTPA [5].

The PPV of 0.3208 indicates that approximately 32% of patients with elevated D-dimer levels actually had PE. In contrast, the NPV of 1.0 indicates that patients with low D-dimer levels were accurately identified as not having PE. These findings emphasize the utility of D-dimer as a rule-out test, minimizing unnecessary CTPA scans in patients with low D-dimer levels and low clinical suspicion for PE [6]. This approach could optimize resource utilization, reduce patient exposure to radiation, and improve overall diagnostic efficiency [7].

Previous studies have highlighted the importance of integrating D-dimer testing with clinical assessment and imaging for accurate PE diagnosis. For instance, the ADJUST-PE study proposed age-adjusted D-dimer cutoff levels to enhance diagnostic accuracy in older patients, reducing false positives [8]. Similarly, the Wells criteria, combined with D-dimer testing, have been validated as effective clinical tools for stratifying PE risk and guiding imaging decisions [9,10]. These approaches underscore the need for a balanced diagnostic strategy that incorporates both biomarkers and clinical judgment [11]. The findings suggest that while D-dimer is a valuable screening tool due to its high sensitivity and NPV, its low specificity means it should be used in conjunction with other diagnostic methods, such as CTPA, to confirm PE [12,13]. This reinforces the utility of D-dimer in ruling out PE but highlights its limitations in positive diagnosis [14,15].

Comparative analysis with similar studies reveals consistent findings. For example, a study by Righini et al. found that combining clinical pretest probability with D-dimer testing could effectively exclude PE in a significant number of patients, thereby reducing the need for unnecessary imaging [16]. Another study by Wells et al. validated the Wells criteria combined with D-dimer testing as an efficient method for PE risk stratification [17]. These studies, along with the present research, support a multifaceted approach to PE diagnosis, integrating D-dimer levels, clinical evaluation, and imaging [18].

The study categorized PE cases into four distinct groups based on the location and severity of the embolism: Category 0: no PE, Category I: peripheral PE, Category II: PE in lobar arteries, and Category III: central embolisms in the pulmonary trunk or arteries.

The analysis of D-dimer levels across these categories reveals varying degrees of correlation between D-dimer levels and the location of PE [19]. Category III: central embolisms in the pulmonary trunk or arteries: The patients in this category exhibited significantly higher mean D-dimer levels at 7.1 mg/L, suggesting a potential correlation between elevated D-dimer levels and the severity of embolism in central pulmonary arteries. Category I: peripheral PE: The mean D-dimer level for this group was 4.3 mg/L, slightly higher than that in other categories but not significantly different enough to establish a strong correlation with the location of the embolism. Category II: PE in lobar arteries: The patients in this group had a mean D-dimer level of 3.6 mg/L, identical to that observed in patients with no PE (Category 0). This finding indicates that D-dimer levels alone may not differentiate between the absence of PE and lobar PE. Category 0: no PE: As mentioned, the mean D-dimer level in this group was 3.6 mg/L, similar to that observed in Category II, where patients had PE in lobar arteries.

Interestingly, these results differ from those reported by Kubak et al., who found that elevated D-dimer levels were more strongly correlated with the location of embolism, particularly in more central locations [20]. The discrepancy between our findings and those of Kubak et al. may be attributed to differences in study design, sample size, and patient population. Another factor that could contribute to the variation in results is the half-life of D-dimer [21]. D-dimer levels decrease over time after the initial thrombotic event, which might have affected the levels measured during our study, particularly if the timing of the blood draw varied between patients. In this study, all D-dimer samples were collected prior to conducting the CTPA. However, due to the varied presentations of the patients, the exact timing of the thrombotic events could not be pinpointed to a specific incident. This temporal decline in D-dimer concentration could obscure the correlation with the specific location of the embolism, especially in cases where the embolism had been present for a longer period before diagnosis.

These findings underscore the importance of interpreting D-dimer levels within the broader clinical context. While elevated D-dimer levels indicate a thrombotic process, they are not specific enough to determine the exact location of PE without further imaging. The significantly higher D-dimer levels observed in central PE (Category III) highlight the potential severity of the condition and the need for prompt diagnostic confirmation and intervention.

Limitations

The retrospective nature of this study and its reliance on hospital records may introduce biases and limit the generalizability of the findings. Additionally, although adequate for initial analysis, the sample size warrants larger prospective studies to confirm these results. Variations in D-dimer assay methods across different laboratories could also affect the consistency and applicability of the findings. Additionally, the absence of assessment for deep vein thrombosis (DVT), which can also increase D-dimer levels, may have affected our ability to fully interpret the relationship between D-dimer levels and PE.

## Conclusions

D-dimer testing is valuable as a screening tool for PE due to its high sensitivity and NPV. However, the poor correlation of D-dimer levels with specific locations of PE, except in central embolism, requires additional diagnostic investigations, such as CTPA, for the actual diagnosis and extent of PE. Generally, elevated levels of D-dimer indicate a thrombotic process but do not highly correlate with the anatomic site of PE except in central embolism. In particular, in central PE, where the embolism is in the pulmonary trunk or its main pulmonary arteries, the levels of D-dimer were significantly higher, indicating that higher D-dimer levels might correlate with the severity of embolism in these central locations. D-dimer levels are of value as an initial test in suspected PE but require interpretation in the context of clinical findings and imaging to arrive at an accurate diagnosis for appropriate management.
